# ﻿On *Cytheridellawhitmani* sp. nov. (Crustacea, Ostracoda) from Cape Cod (Massachusetts, USA), with a reappraisal of the taxonomy of the genus

**DOI:** 10.3897/zookeys.1224.135458

**Published:** 2025-02-03

**Authors:** Koen Martens, Nadiny Martins de Almeida, Michael Shribak, Janet Higuti, Isa Schön

**Affiliations:** 1 Royal Belgian Institute of Natural Sciences, Freshwater Biology, Vautierstraat 29, B-1000 Brussels, Belgium; 2 Department of Biology, Ghent University, K.L. Ledeganckstraat 35, B-9000 Ghent, Belgium; 3 Graduate Program in Ecology of Inland Water Ecosystems (PEA), Department of Biology (DBI), Centre of Biological Sciences (CCB), State University of Maringá (UEM), Av. Colombo, 5790, CEP 87020-900, Maringá, PR, Brazil; 4 Marine Biological Laboratory, Woods Hole, MA, USA; 5 Centre of Research in Limnology, Ichthyology and Aquaculture (Nupélia), Centre of Biological Sciences (CCB), State University of Maringá (UEM), Av. Colombo, 5790, CEP 87020-900. Maringá, PR, Brazil; 6 Centre of Environmental Sciences (CMK), University of Hasselt, 3590 Diepenbeek, Belgium

**Keywords:** African species, caudal ramus, hemipenis, invasive species, valve ornamentation

## Abstract

*Cytheridellawhitmani* Martens, **sp. nov.** is described from lakes on Cape Cod (MA, USA). The species differs from its congeners mainly by the shape of the female carapace and by the morphology of the hemipenis, especially of the distal lobe and the copulatory process. The literature on the genus is reviewed and the synonymy of the fossil *Cytheridellaboldii* Purper, 1974 with the type species *C.ilosvayi* Daday, 1905, both described from South America, is confirmed. The status of *Cytheridellaamericana* (Furtos, 1936) is reverted to that of “uncertain species”. Beside the type species and the new species, the genus currently includes only three further species from Africa: *C.monodi* Klie, 1936, *C.damasi* Klie, 1944 (with synonym *C.chariessa* Rome, 1977), and *C.tepida* Victor, 1987. The morphology of the new species is discussed in comparison with the congeneric species, especially regarding the valve ornamentation, the structure and function of the third thoracopod, the hemipenis and the caudal ramus. It is suggested that *C.whitmani* is a recent invasive species in the lakes of the Cape Cod peninsula. Its occurrence at northern latitudes is unexpected, as its congeneric species are consistently (sub-) tropical.

## ﻿Introduction

Non-marine ostracods (small, bivalved crustaceans) occur on all continents except Antarctica, and in most aquatic and (semi-) terrestrial environments (Smith et al. 2015). The knowledge on the diversity of non-marine ostracods on different continents and in different zoogeographical regions is highly unequal and this mostly for historical reasons. In Europe, living non-marine ostracods have consistently received much more taxonomic and ecological attention over the past one and a half century than in North America, whereas for most ostracod groups (genera, subfamilies), the North American fauna is more speciose than the European one (Martens et al. 2008; Meisch et al. 2019).

The non-marine ostracod fauna of Massachusetts has been investigated by Haldeman (1842), Cushmann (1905, 1907), Sharpe (1908, 1910), and Furtos (1935). A total of 22 species has thus far been reported by these authors from Massachusetts, 13 of these from Cape Cod (Table 1). Especially the more extensive survey of Furtos (1935) is of interest here, as most species she described or reported on are from localities on Cape Cod, the peninsula dealt with in the present study.

**Table 1.** Ostracod species reported from Massachusetts (M) and from Cape Cod specifically (CC) in the literature. Note: *Cyprisscabra* Haldeman, 1842 is here considered an uncertain species, as was already foreshadowed by Furtos (1935), and is not listed here. Martens et al. (2023) reported a sexual population of *Cypridopsisvidua* from Cape Cod.

Genus and species	Authority	Cushman, 1905	Cushman, 1907	Sharpe, 1908	Sharpe, 1910	Furtos, 1935	Present Paper
*Heterocyprisincongruens*	(Ramdohr, 1808)	M		M			
Cyprinotussyn.?americanus	Cushman, 1905	M					
*Spirocyprispassaica*	Sharpe, 1903		M	M			
*Eucyprisvirens*	(Jurine, 1820)		M	M			
*Bradleystrandesiafuscata*	(Jurine, 1820)		M				
*Bradleystrandesiareticulata*	(Zaddach, 1844)		M	M			
*Bradleystrandesiasplendida*	(Furtos, 1933)					M	
*Cypridopsisvidua*	(O.F. Müller, 1776)		M				
*Cypriaexculpta*	(Fischer, 1855)		CC				
*Cypriaobesa*	(Sharpe, 1897)				CC		
*Cypriapalustera*	(Furtos, 1935)					CC	
*Physocypriaposterotuberculata*	(Furtos, 1935)					CC	
*Physocypriaglobula*	(Furtos, 1933)					CC	
*Cyclocyprisforbesi*	(Sharpe, 1897)					CC	
*Cyclocypriscruciata*	Furtos, 1935					CC	
*Candonacandida*	(O.F. Müller, 1776)		M				
*Candonadecora*	(Furtos, 1933)					CC	
*Fabaeformiscandonacaudata*	(Kaufmann, 1900)					CC	
*Pseudocandonaannaeseptentrionalis*	(Furtos, 1935)					CC	
*Pseudocandonaelliptica*	(Furtos, 1933)					CC	
*Pseudocandonapunctata*	(Furtos, 1933)					CC	
*Darwinulastevensoni*	(Brady & Robertson, 1870)					CC	
*Cytheridellawhitmani*	this study						CC

*Cytheridella* Daday, 1905 belongs to the family Limnocytheridae Sars, 1925, subfamily Timiriaseviinae Mandelstam, 1960 (at this stage we do not follow Tanaka et al. (2021) in raising this subfamily “to a higher taxonomic level”) and tribe Cytheridellini Danielopol & Martens, 1989 (in Danielopol et al. 1989). Its type species, *C.ilosvayi* Daday, 1905, originally described from Paraguay, turned out to be one of the most common inhabitants of Neotropical water bodies (Higuti et al. 2010; Conceição et al. 2020). The morphology of *C.ilosvayi* has been extensively studied over the past decade. For example, Wrozyna et al. (2014, 2016, 2018, 2019) performed quantitative valve outline analyses in search of discrete morphotypes, with implications for ontogeny and zoogeography. Danielopol et al. (2018) and Lord et al. (2020) formalised the different types of sieve-type pore canals and demonstrated their relevance for ostracod taxonomy. Danielopol et al. (2023) compiled an extensive diagnosis of *C.ilosvayi*, mostly based on valve morphology, in comparison with several fossil *Cytheridella* species. Here, we describe a new extant species of the genus *Cytheridella* found in several lakes (locally referred to as “ponds”) on Cape Cod and re-asses the validity of the known recent species.

## ﻿Materials and methods

### ﻿Study area

**Figure 1.**
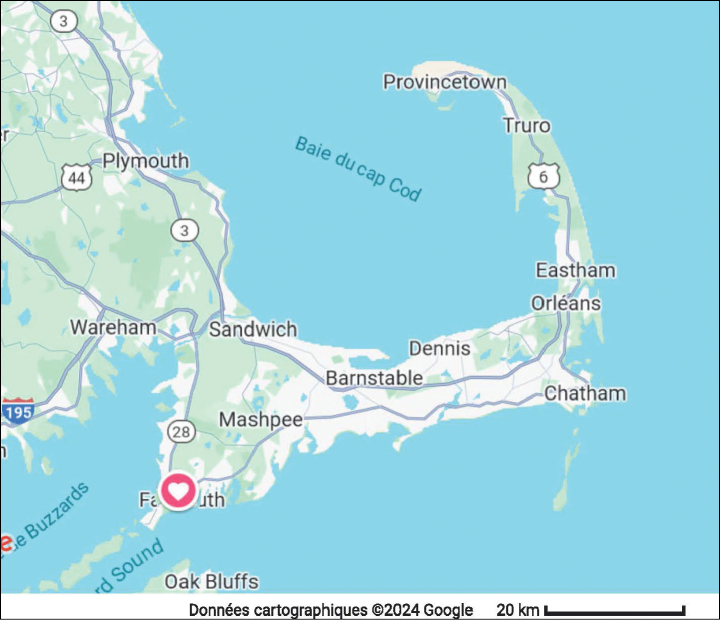
Map of Cape Cod (MA, USA). The symbol indicates the position of Grews Pond (Falmouth), the type locality of *Cytheridellawhitmani*.

Samples in the present study were taken in the south-western half of Cape Cod. This peninsula extends into the Atlantic Ocean at the eastern shore of North America, looking like a crooked arm (Fig. 1). It has an east-west oriented basal part and a south-north oriented distal part. It is a sandy peninsula, mostly formed during the last ice age. By ca 18,000 years ago, the ice sheets had retreated past Cape Cod. The resulting landscape, especially of the basal part, is one littered with Holocene (Kettle) lakes of varying shapes, depth and surface sizes (Fisher and Leatherman 1987).

### ﻿Sampling and sample treatment

Semi-quantitative samples were taken with a rectangular hand net (mesh size 160 µm), using waders, between 10 cm and 1.5 m deep by moving the net amongst vegetation and over the bottom sediment. All available habitats (exposed sand and gravel beaches, submerged weed beds, emerging macrophyte stands and accumulated debris, fallen leaves etcetera) at the public access areas (boat ramps) of each lake were sampled. In situ measurements were taken with portable meters of water electrical conductivity (Greisinger 480846), pH and temperature (Ebro PHX800). Ostracods were sorted in the laboratory from the total samples under a stereo-binocular microscope (Leica) and were stored in Eppendorf tubes in 100% ethanol, buffered with borax to prevent decalcification of the valves.

Soft parts were separated from the valves using dissection needles and were put in a drop of glycerine for the dissection of the appendages. The dissection was covered with a coverslip and sealed with transparent nail polish. Valves were stored dry in micropaleontological slides. Drawings of soft parts were made using a camera lucida (Olympus U-DA) attached to the microscope (Olympus CX-41). Carapace and valves were illustrated and measured using Scanning Electron Microscopy (SEM, Fei Qanta 200 ESEM, in the Royal Belgian Institute of Natural Sciences, Brussels, Belgium) in different views and details. The hemipenis of the new species was also illustrated using the polychromatic polarisation microscope. The “Polscope” uses polarisation interference colours to show details of tissues that would otherwise be invisible. It was invented by Michael Shribak (Shribak 2014, 2015) at the Marine Biological Laboratory (MBL, Woods Hole, MA, USA). The Polscope set-up used here consisted of a microscope Olympus IX81, with objective lens magnification 20 × and total magnification 20 × 16, and a colour CCD camera Olympus DP73. The only previous use of this technology for imaging ostracods was in Martens et al. (2023).

Chaetotaxy of the limbs largely follows the model proposed by Broodbakker and Danielopol (1982). Higher taxonomy of the Ostracoda follows Horne et al. (2002), Meisch et al. (2019, 2024) and for the Timiriaseviinae, Danielopol et al. (2018). Repository: Royal Belgian Institute of Natural Sciences, Brussels, Belgium (**RBINS**; general inventory number IG34899, specimens numbers INV323000-3230021).

### ﻿Abbreviations used in text and figures

#### ﻿Valves and carapaces

**Cp** carapace

**CpD** carapace in dorsal view

**CpRL** carapace in right lateral view

**CpV** carapace in ventral view

**H** height

**il** inner list

**L** length

**LV** left valve

**LVi** left valve in internal view

**ol** outer list

**RV** right valve

**RVi** right valve in internal view

**W** width

#### ﻿Limbs

**A1** antennula

**A2** antenna

**cop** copulatory process on Hp

**CR** caudal ramus (“organ fourchu” in female)

**d, d_p_, e, f, h2, h3** claws and setae on T2 and T3

**dej** ductus ejaculatorius in copulatory process

**DL** distal lobe of Hp

**En1–En4** endopodite segments 1–4 of T1–T3

**Hp** hemipenis

**Md** mandibula

**MdPalp** mandibular palp

**Mx1** maxillula

**T1** first thoracopod

**T2** second thoracopod

**T3** third thoracopod

**Y, Ya** aesthetascs on A2 and A1 respectively

## ﻿Results

**Class Ostracoda Latreille, 1802**

**Subclass Podocopa G.O. Sars, 1866**

**Order Podocopida G.O. Sars, 1866**

**Suborder Cypridocopina Baird, 1845**

**Superfamily Cytheroidea Baird, 1850**

**Family Limnocytheridae Sars, 1928 (fide Danielopol et al. 2018)**

### ﻿Subfamily Timiriaseviinae

Taxon classificationAnimaliaPodocopidaLimnocytheridae

Mandelstam, 1960

5EACDEFF-5615-5DCA-93FE-75444064394E

Metacypridinae Danielopol, 1960 (fide Colin and Danielopol 1978). Syn.

### ﻿Tribe Cytheridellini

Taxon classificationAnimaliaPodocopidaLimnocytheridae

Danielopol & Martens, 1989

3E3088EC-5508-5D2A-8189-CD472180D8C8

#### Allocated genera.

*Cytheridella* Daday, 1905; *Gomphocythere* Sars, 1924. Note: the genus *Gomphodella* De Deckker, 1981 is now lodged in the tribe GomphodelliniDanielopol et al. 2018.

### ﻿Genus Cytheridella

Taxon classificationAnimaliaPodocopidaLimnocytheridae

Daday, 1905

225BAECD-DB8A-5DA1-980B-C468560E7D45

Onychocythere Tressler, 1939 (fide Pinto and Sanguinetti 1962). Syn.

#### Type species.

*Cytheridellailosvayi* Daday, 1905.

Syn.: *Metacyprisometepensis* Swain & Gilby, 1964 (fide Purper 1974; Martens and Behen 1994).

Syn.: *Onychocytherealosa* Tressler, 1939 (fide Purper 1974; Cohuo et al. 2017).

Syn.: *Gomphocythereargentinensis* Ferguson, 1967 (fide Karanovic 2009).

Syn.: *Cytheridellaboldii* Purper, 1974 (fide Danielopol et al. 2018).

#### Diagnosis

**(partly derived from the extensive analysis of Danielopol et al. 2023).**Cp largely sexually dimorphic. Males: CpV and CpD laterally rather flattened, with greatest width slightly behind the middle, both anterior and posterior sides pointed. Females: CpD and CpV with highly developed brood chamber, occupying 2/3 of the posterior part of the Cp, posterior margin almost straight, anterior margin pointed. In both sexes with well-developed lateral sulci and external valve surfaces heavily ornamented, with pits, rimmed pores with setae, and, especially anteriorly and posteriorly, with long and stiff setae and pores on conical elevations with setae (*Porenwarzen*). In inner views, both valves with well-developed anterior and posterior selvages, largely inwardly displaced; anterior calcified inner lamella of both valves set with two connected rows of long and fine cuticular filaments (setulae). Hinge adont. Central Muscle Scars consisting of a vertical row of four scars.

A1 with second segment bearing a long seta on the proximo-ventral side; penultimate segment fully or partly fused (segments 4+5); one of dorso-apical setae on this segment shaped as a trident. A2 with three distal claws. T1 and T2 with segment En4 fused with end claw. T3 a reflexed “cleaning limb”, with segment 4 not fused with end claw, seta h3 a spine. In females, with elongated CR (“organ fourchu”), with bifurcated tip. Hp with DL hinging on basal part, copulatory process coiled, short or (very) long. In males, CR simple but robust setae.

#### Other (African) species.

*C.damasi* Klie, 1944 (Congo, syn.: *C.chariessa* Rome, 1977 (Congo, in Rome and De Deckker 1977, fide Karanovic 2009)); *Cytheridellamonodi* Klie, 1936 (Cameroon) *Cytheridellatepida* Victor, 1987 (Nigeria).

#### Remark.

*Cytheridellaamericana* (Furtos, 1936) Danielopol (1981 in Colin and Danielopol 1980) from Yucatan (Mexico) is here considered an uncertain species (see below).

### ﻿ Cytheridella whitmani

Taxon classificationAnimaliaPodocopidaLimnocytheridae

Martens sp. nov.

A7FDC355-747E-5560-B173-4CC49B3DA237

https://zoobank.org/2560DC0D-F302-47F3-BE20-6BCB7C5F3476

Figs 2 , 3 , 4 , 5 , 6 , 7 , 8 , 9 , 10 , 11 , 12

#### Type material.

***Holotype*** • 1 ♂ (adult); dissected and stored on a permanent microscopic slide and valves stored dry in a micropalaeontological slide (nr INV323000). ***Allotype*** • 1 ♀ (adult); dissected and stored as the holotype (nr INV323001). ***Paratypes*** • 3 ♂♂ adult Cp used for SEM (nrs INV323002-323004). 1 ♂ dissected and stored as the holotype (nr INV323005). 3 ♀♀ adult Cp used for SEM (nrs INV323006, INV323008, INV323010,). 1 ♀ dissected and stored as the holotype (nr INV3230014). Thirty ♀♀ and ♂♂ in EtOH (INV3230021).

#### Type locality.

USA • Massachusetts, Cape Cod, Grews Pond, Goodwill Park, Falmouth. Coordinates: N: 41.5696816, W: 70.6146054. Altitude: 5 m a.s.l. Collected on 27 July 2023. Leg.: Koen Martens and Isa Schön. Measurements at the time of collecting: Electrical Conductivity: 49 µS/cm, pH: 7.4, Water Temperature: 28 °C (holotype, allotype, and paratypes are all from the type locality).

#### Other localities on Cape Cod

**(details on ecology will be provided elsewhere). *Woods Hole***: Miles Pond. ***Falmouth***: Mares Pond, Deep Pond, Coonamessett Pond. ***Mashpee***: Wakeby Pond, Peters Pond, Pemlico Pond. ***Barnstable***: Lorells Pond, Snake Pond, Mystic Pond, Middle Pond, Hamblin Pond, Shubael Pond, Wequaquet Pond, Dennis Pond. ***Bourne***: Flax Pond. Sandwich: Laurence Pond, Spectacle Pond. ***Yarmouth***: Long Pond.

#### Etymology.

The species is named after Dr Charles Otis Whitman (1842–1910), professor at the University of Chicago, and the first director of the Marine Biological Laboratory (MBL) at Woods Hole (Ma, USA), after whom one of the present Research Centres at MBL and a series of fellowships are named (https://en.wikipedia.org/wiki/Charles_Otis_Whitman). The name is a noun in the genitive singular.

#### Diagnosis.

Cp as typical for the genus and in dorsal view most similar to the type species, but significantly smaller. Valves in inner view both with largely inwardly displaced selvage, especially in the poster-ventral corner of the RV. Posterior flanges of both valves on the inner side set with a series of rimmed pores, each bearing a simple seta. A1 with ventro-apical seta strong and claw-like. Mx1 palp apically with four claws and one seta. T3 a cleaning limb, with endopodal segment 4 fused with terminal claw, seta h3 a spine. Hp with DL elongated, sub-rectangular with bluntly pointed ventro-distal edge, and a long narrow, coiled copulatory process, distally pointed.

#### Description.

**Figure 2.**
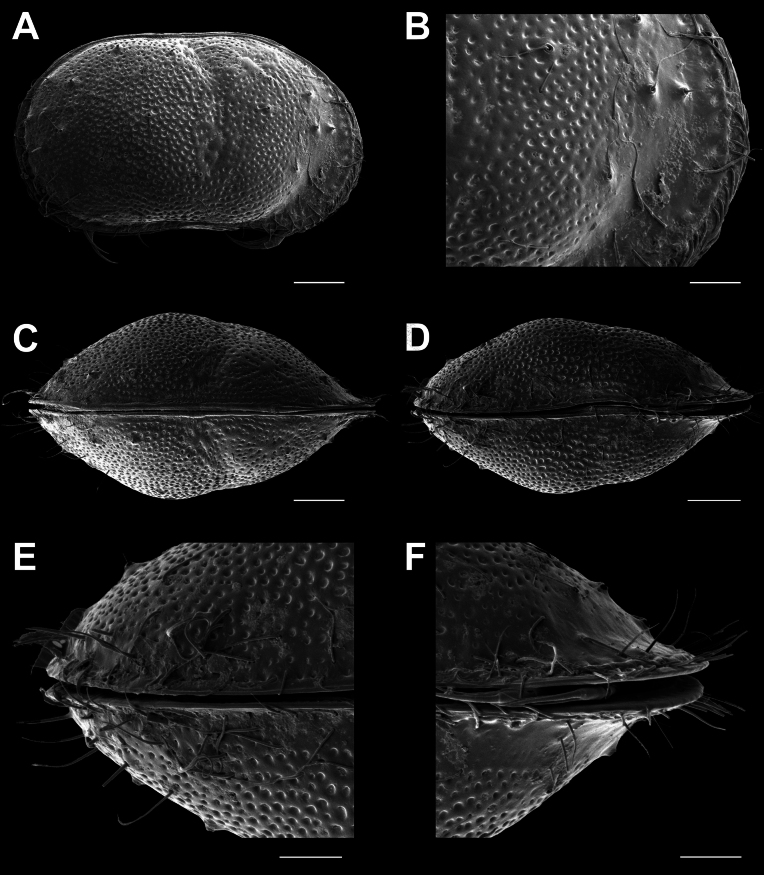
*Cytheridellawhitmani*, male **A**CpRL (INV323004) **B**CpRL, detail of anterior part (INV323004) **C**CpD (INV323002) **D**CpV (INV323003) **E**CpV, detail of posterior part (INV323003) **F**CpV, detail of anterior part (INV323003). Scale bars: 100 μm (**A, C, D**); 50 μm (**B, E, F**).

**Male.**CpRL (Fig. 2A, B) view rectangular, with widely rounded posterior and anterior margins, the latter slightly ventrally produced; dorsal margin straight for more than half of its length, ventral margin sinuous slightly anteriorly to the middle; with in both valves a clear lateral dorso-medial sulcus (reaching from dorsal side to more than half the height of the valves) and an antero-ventral sulcus (reaching from ventral side to more than half the height of the valves). CpD (Fig. 2C) and CpV (Fig. 2D–F) with pointed anterior and posterior margins and unevenly rounded lateral sides, the latter interrupted by the lateral sulci, greatest width situated slightly behind the middle. External valve surface heavily ornamented, set with circular and longitudinal pits and rimed pores, the latter especially anteriorly and posteriorly with long and stiff setae (*Porenwarzen*).

**Figure 3.**
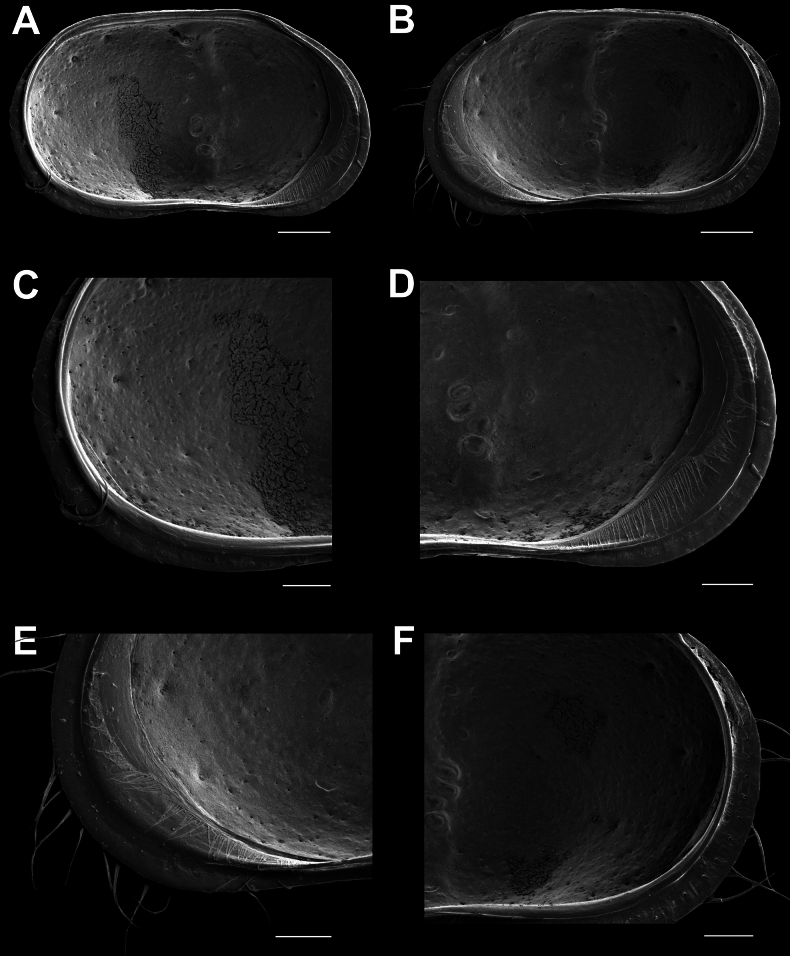
*Cytheridellawhitmani*, male **A**LVi (INV323005) **B**RVi (INV323005) **C**LVi, detail of posterior part (INV323005) **D**LVi, detail of anterior part (INV323005) **E**RVi, detail of anterior part (INV323005) **F**RVi, detail of posterior part (INV323005). Scale bars: 100 μm (**A, B**); 50 μm (**C–F**).

RVi (Fig. 3B, E, F) with shape as for the CpRL, with straight dorsal and slightly sinuous ventral sides, both anterior and posterior margins widely rounded; with well-developed selvage widely inwardly displaced along anterior and posterior margins, especially in the postero-ventral part; this part of the flange with a circular line of rimmed pores with setae.

LVi (Fig. 3A, C, D) almost symmetrically to RV, but with selvage less inwardly displaced, especially in the postero-ventral part.

**Figure 4.**
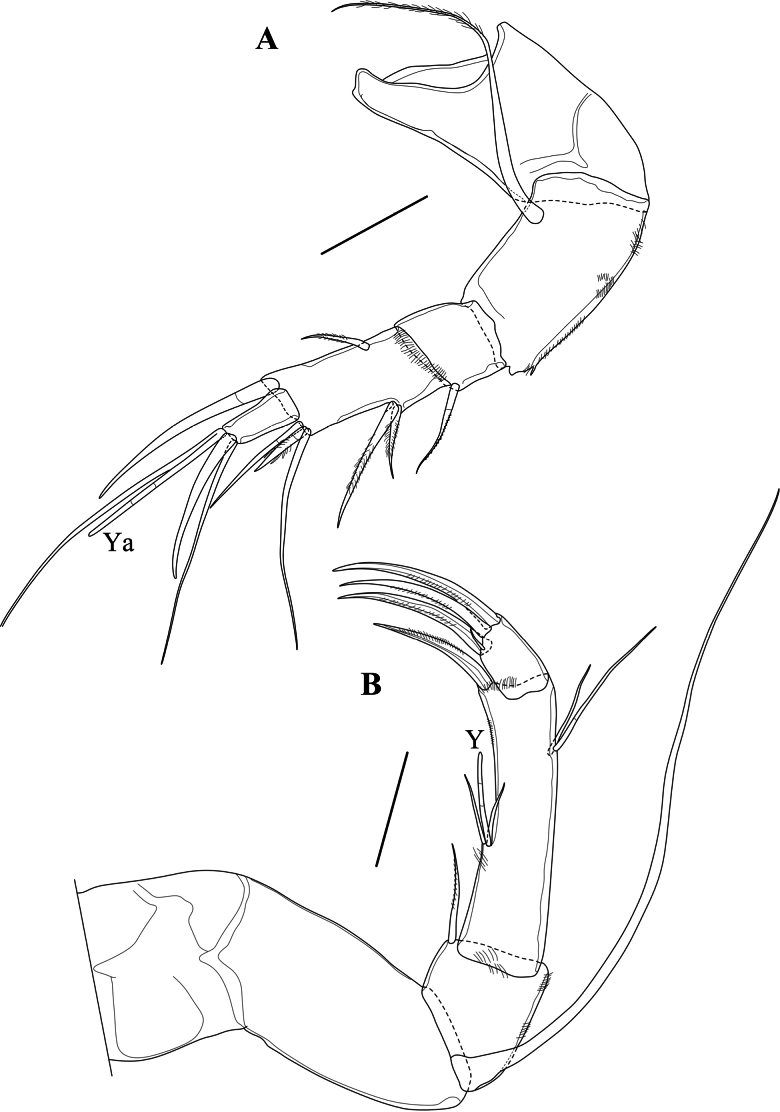
*Cytheridellawhitmani*, holotype male **A**A1 (INV323000) **B**A2 (INV323000). Scale bars: 50 µm.

A1 (Fig. 4A). Five-segmented. First segment slightly longer than wide. Second segment slightly shorter than the first one, with one long ventral and distally plumose seta, sub-basically inserted and reaching tip of penultimate segment. Third segment sub-quadrate, with a single dorso-apical seta reaching mid-length of fourth segment. Fourth segment (fusion of ancestral 4^th^ and 5^th^ segments) approximately twice as long as basal width. Setation of ancestral fourth segment: two unequal dorso-medial setae, one ventro-medial seta approximately as long as the shortest dorso-medial seta. Setation of ancestral fifth segment: three dorso-apical setae: one long, one short and one approximately one third the length of the long one; this latter seta broad and distally with two spines, almost looking like a trident, consisting of apical point and two subapical spines) and one seta of intermediate length, slightly longer than half the length of the long seta; further with one long ventro-apical claw-like seta. Fifth (terminal) segment approximately 1.5 times as long as the basal width, apically with aesthetasc Ya and its longer accompanying seta, fused at the base with that of the Ya, one long seta, almost as long as the accompanying seta of Ya and a shorter, but stout claw.

A2 (Fig. 4B). Protopodite two-segmented. First segment short. Second segment approximately twice as long as basal width. Exopodite a long, one-segmented spinneret seta, reaching beyond tips of end claws. Endopodite three-segmented. En1 skewed rectangular, approximately as long as basal width, with a short ventro-apical seta, reaching halfway along the second segment; dorso-apically with some pseudochaeta. En2 approximately five times as long as basal width; mid-ventrally with a short aesthetasc Y, flanked on each side by a subequal seta; dorsally with two sub-apical setae, one approximately half the length of the other and ventro-apically with a large claw, more than two times the length of the third segment. En3 (terminal segment) small, skewed sub-quadrate, apically with three large and subequal pectinate claws.

**Figure 5.**
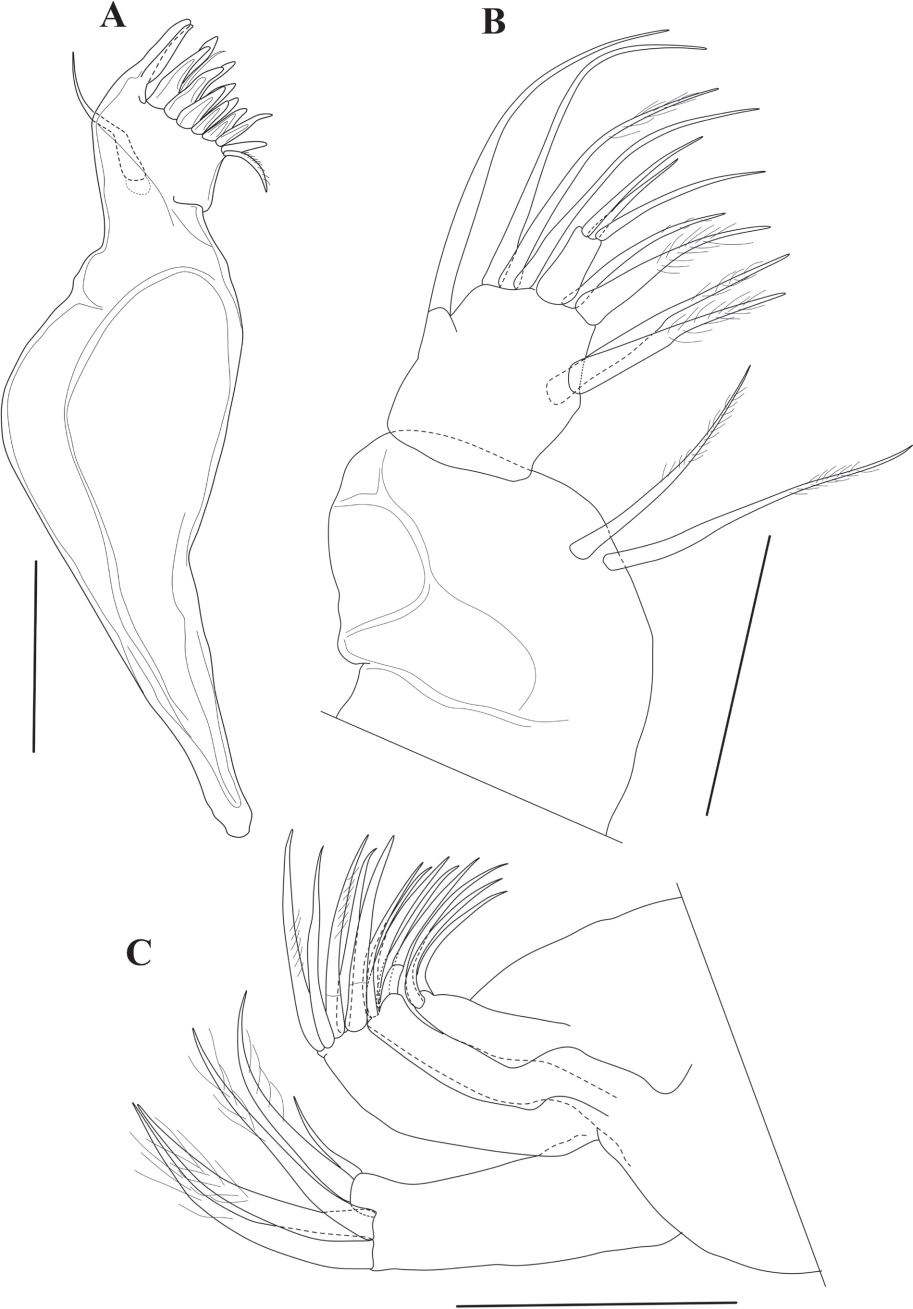
*Cytheridellawhitmani*, holotype male **A**Md (INV323000) **B**Md-palp (INV323000) **C**Mx1 (INV323000). Scale bars: 50 µm.

Md coxa (Fig. 5A) long and curved, apically with eight strong teeth, some doubled, interspaced with thin setae, ventro-apically with a short, reflexed plumose seta; sub-apically with a long and stout seta, not reaching the tips of the claws.

MdPalp (Fig. 5B) three-segmented. First segment ventrally set with two large, plumose sub-apical setae, one approximately 3/4 the length of the other, and a respiratory plate (not illustrated). Second segment (fusion of two segments) mid-ventrally with two large and stout setae, almost equally long and plumose in the distal half; mid-dorsally with one long and smooth seta, reaching beyond all other setae, ventro-apically with one long and stout seta, plumose in the distal half and one short, thin and largely smooth seta; dorso-apically with a bunch of three long, sub-equal setae, mostly smooth. Third (terminal) segment very small, approximately twice as long as the basal width; with three apical setae, one long, one of intermediate length and one shorter than the other two.

Mx1 (Fig. 5C) consisting of a basis, a large respiratory plate (not illustrated), three endites and a one-segmented palp. First endite with three subequal, slender setae. Second endite with five subequal, claw-like setae. Third endite with five claw-like setae, four large and one half the size of the others. Palp with four long claws, distally plumose and one small smooth seta, approximately half the length of the claws. Respiratory plate (exopodite – not illustrated) with approximately 16 plumose rays.

**Figure 6.**
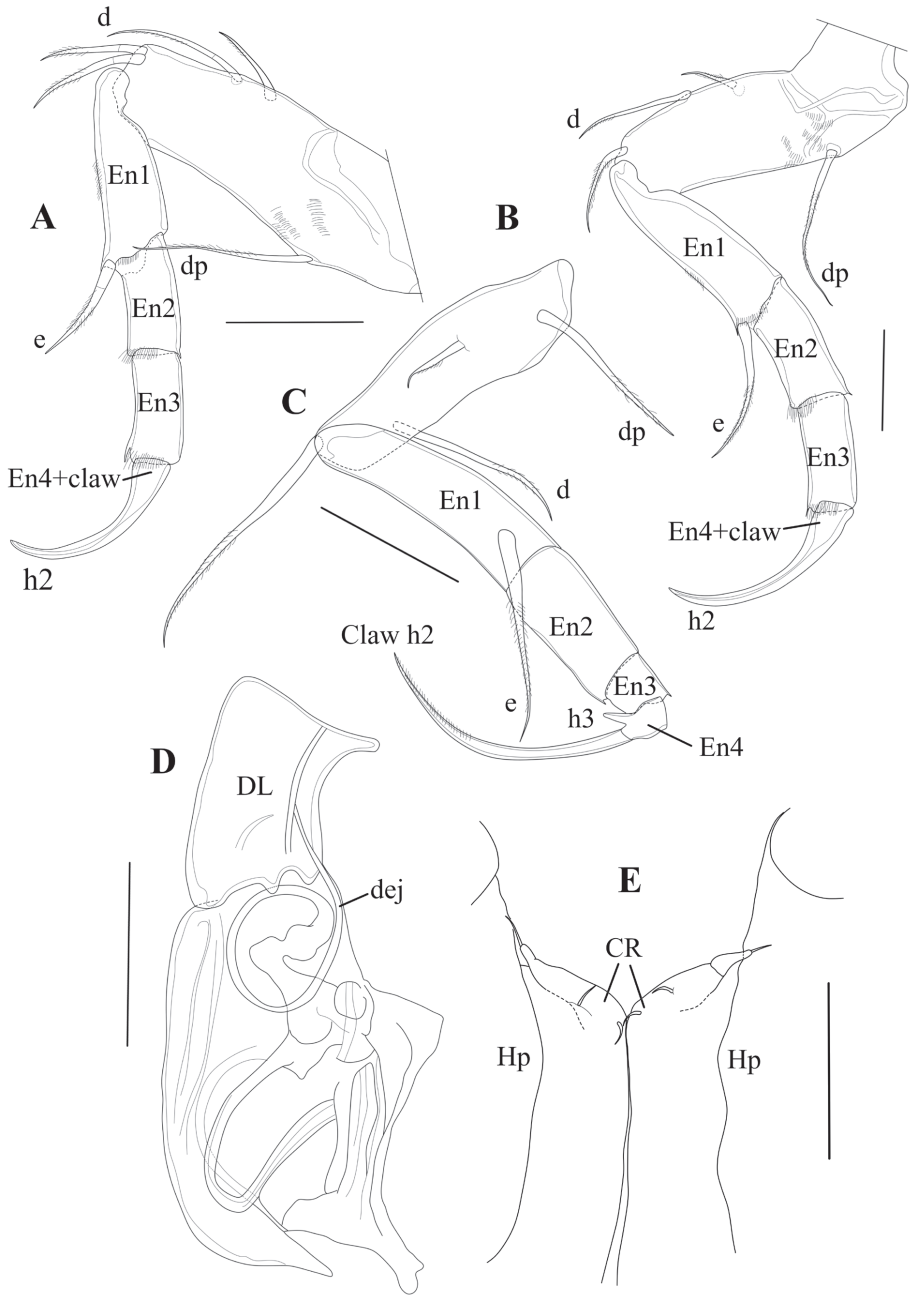
*Cytheridellawhitmani*, holotype male **A**T1 (INV323000) **B**T2 (INV323000) **C**T3 (INV323000) **D**Hp (INV323000) **E**CR in between two Hp. (INV323015). Scale bars: 50 µm.

T1 (Fig. 6A) a four-segmented walking leg. Basal segment (Basis) long and broad, with one long ventral seta dp, almost reaching distal tip of segment, two short, subequal dorso-apical setae and a two mid-dorsal seta, the most distally inserted one approximately twice as long as the proximal one and reaching distal tip of segment. Segment En1 with one stout ventro-apical seta (e seta) reaching tip of En2. Segment En2 with length approximately 1.5 times basal width and without setae. En3 with length similar to that of second endopodal segment and also without setae; apically with one long and curved distal claw (h2), distally pectinate and basally incorporating segment En4.

T2 (Fig. 6B) also a four-segmented walking leg, slightly larger than the first thoracic limb. Basal segment (Basis) with long and thin mid-ventral seta dp, plumose in the distal 2/3 of its length; one short dorso-apical seta and two mid-dorsal setae, the most distally inserted one approximately three times as long as the proximal one and reaching beyond the distal tip of segment. Segment En1 with ventro-apical seta (e seta) approximately as long as the segment itself. En2 and En3 subequal and without setae, distal claw (h2), basally incorporating segment En4, longer and slightly more arched than equivalent claw on T1.

T3 (Fig. 6C) a cleaning leg. Basal segment (basis) elongated, ventrally with a long basal seta dp; apically with a single, long (as long as the segment itself) and smooth seta, mid-dorsally with two setae, the most distally inserted one approximately three times as long as the proximal one and reaching beyond the distal tip of segment with half of its length. En1 the longest endopodal segment, with subapically a long e seta, plumose in its distal third. En2 shorter than En1 by approximately one third, devoid of setae. En3 short, approximately half the length En2 and devoid of setae. En4 even smaller than En3, slightly obliquely inserted on the latter, carrying a long and curved claw h2 (but not fused with it) and a spine-like h3, fitting in a ventro-apical space of En2, thus forming a cleaning pincer.

**Figure 7.**
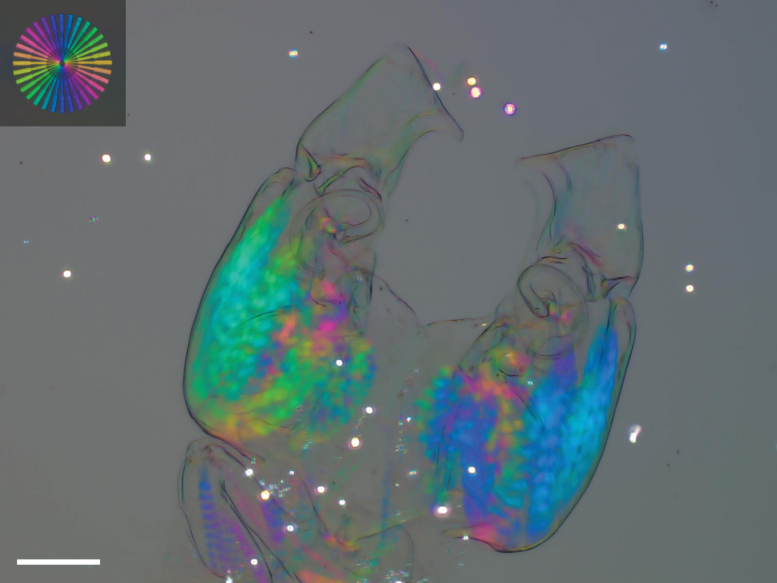
*Cytheridellawhitmani*, male, both Hp (INV323015). Image by polychromatic Polscope. Inset: image of the test target. Crucially, the slow axes of the test target are radial, and their hue can be effectively utilized to determine muscle orientation within the Hp. Scale = 50 µm.

Hp (Figs 6D, 7) with broad, elongated and sclerotised muscular body, comprising three or four main bundles of muscles (see Polscope illustration, Fig. 7), an elongated and sub-rectangular distal lobe (DL), with bluntly pointed ventro-distal edge, with a short seta inserted in the middle of the basal part of the lobe DL, and a long narrow, coiled copulatory process, distally pointed. CR (Fig. 6E) consisting of two stout setae at base of each Hp, but not fused with them.

**Female** (only sexually dimorphic features mentioned).

**Figure 8.**
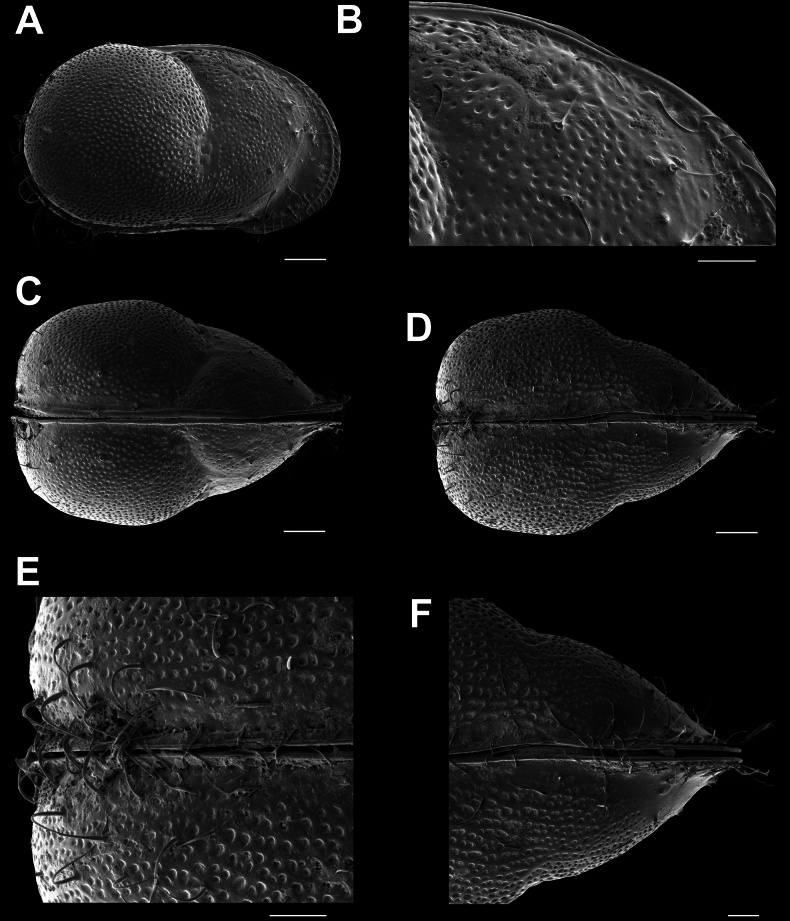
*Cytheridellawhitmani*, female **A**CpRL (INV323008) **B**CpRL, detail of anterodorsal part (INV323008) **C**CpD (INV323006) **D**CpV (INV323007) **E**CpV, detail of posterior part (INV323007) **F**CpV, detail of anterior part (INV323007). Scale bars: 100 μm (**A, C, D**); 50 μm (**B, E, F**).

CpRL (Fig. 8A, B) sub-rectangular, with widely rounded posterior and anterior margins, the latter slightly ventrally produced; dorsal margin not straight, but strongly indented behind the middle at the start of the lateral sulcus, ventral margin slightly sinuous; with a clear dorso-medial sulcus and an anterior ventro-medial sulcus in both valves, as in the male. CpD (Fig. 8C) and CpV (Fig. 8D–F) with pointed anterior margin and posteriorly with highly developed brood chamber, occupying two-thirds of the posterior part of the Cp, posterior margin almost straight. External valve surface heavily ornamented, set with circular and longitudinal pits, rimed pores, especially anteriorly and posteriorly with long and stiff setae in *Porenwarzen*.

**Figure 9.**
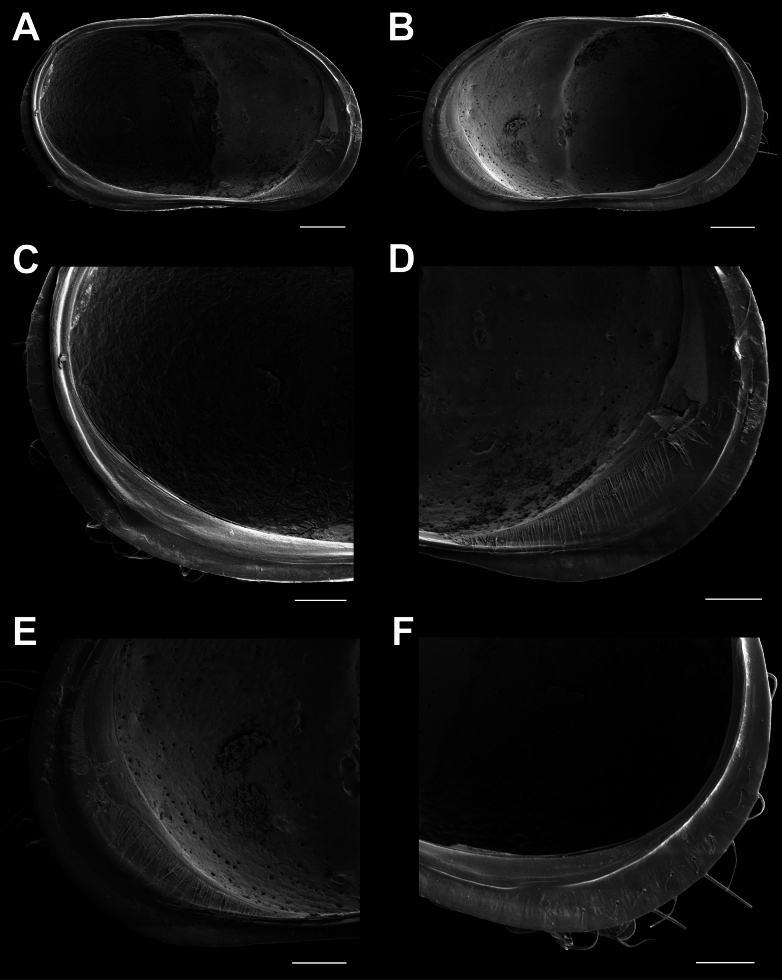
*Cytheridellawhitmani*, female **A**LVi (INV3230014) **B**RVi (INV3230014) **C**LVi, detail of posterior part (INV3230014) **D**LVi, detail of anterior part (INV3230014) **E**RVi, detail of anterior part (INV3230014) **F**RVi, detail of posterior part (INV3230014). Scale bars: 100 μm (**A, B**); 50 μm (**C–F**).

RVi (Fig. 9B, E, F) with shape as for the CpRL, but with straight dorsal and slightly sinuous ventral sides, both anterior and posterior margins widely rounded; well-developed selvage widely inwardly displaced along anterior and posterior margins, especially in the postero-ventral part, this part of the flange with a series of rimmed pores with single setae. Posterior brood pouch most prominent.

LVi (Fig. 9A, C, D) almost symmetrically as the RV, but with selvage slightly less inwardly displaced, especially in the postero-ventral part.

**Figure 10.**
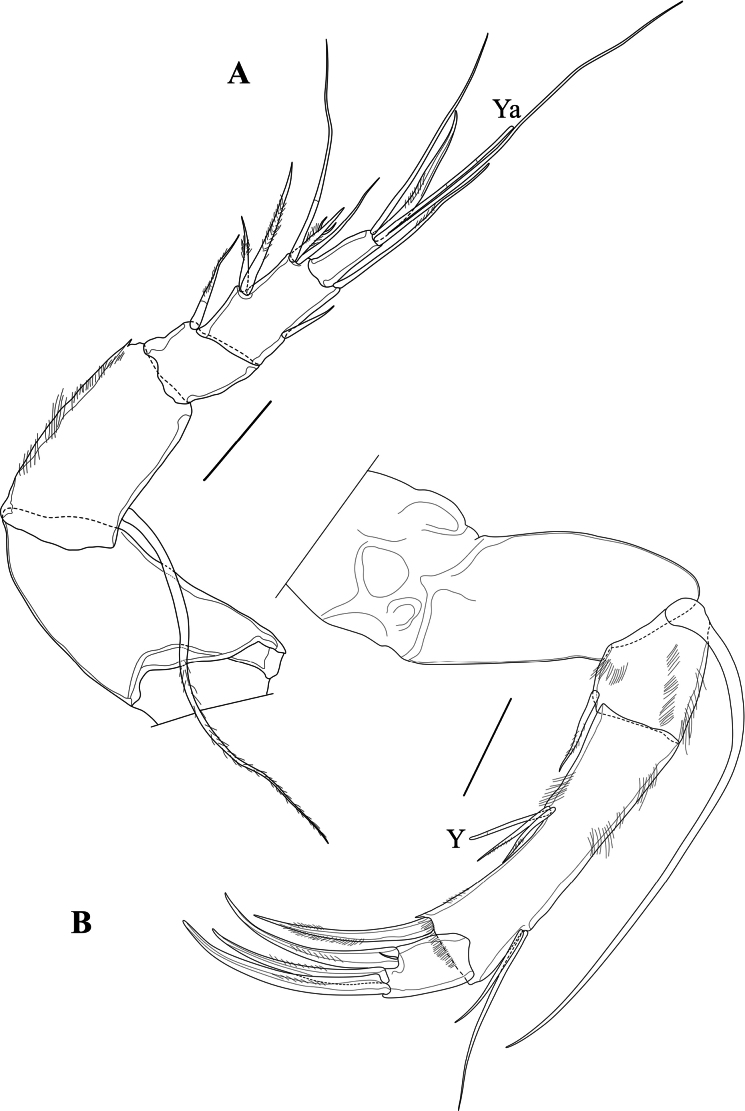
*Cytheridellawhitmani*, allotype female **A**A1 (INV323001) **B**A2 (INV323001). Scale bars: 50 µm.

A1 (Fig. 10A) with “trident” aspect of short dorso-apical seta on fourth segment pronounced (consisting of apical point and two subapical spines); ventro-apical seta in this segment a long seta, not a claw; accompanying seta to aesthetasc Ya on terminal seta twice as long as aesthetasc itself.

A2 (Fig. 10B) with exopodal seta shorter than in the male, not reaching tips of end claws; these three claws more (sub-) equal than in the male.

**Figure 11.**
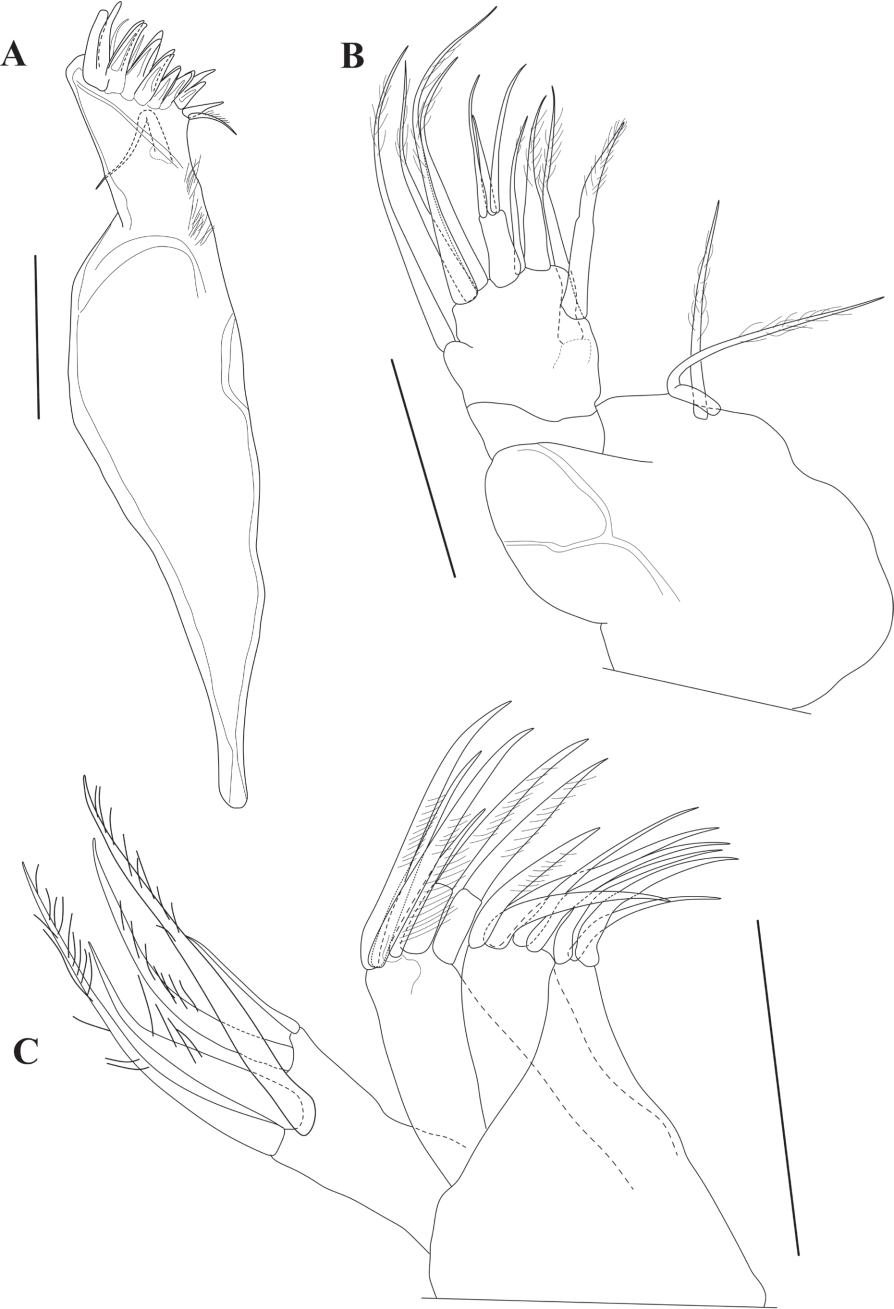
*Cytheridellawhitmani*, allotype female **A**Md (INV323001) **B**Md-palp (INV323001) **C**Mx1 (INV323001). Scale bars: 50 µm.

Md coxa (Md) (Fig. 11A) less sinuous than in the male. Palp (Fig. 11B) with setae in first segment more subequal in length.

Chaetotaxy of endites and palp of Mx1 (Fig. 11C) highly similar to that in the male, but one distal claw on the palp significantly shorter than the three others.

**Figure 12.**
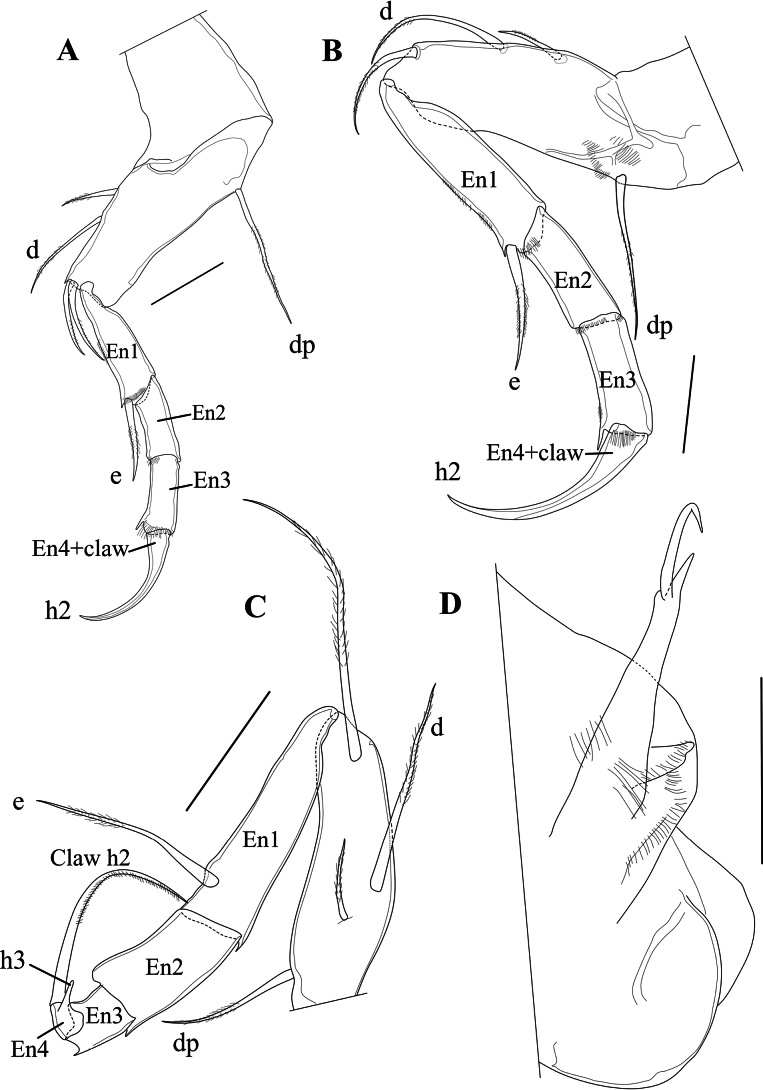
*Cytheridellawhitmani*, allotype female **A**T1 (INV323001) **B**T2 (INV323001) **C**T3 (INV323001) **D**CR and post-abdomen (INV323001). Scale bars: 50 µm.

T1 (Fig. 12A), T2 (Fig. 12B) and T3 (Fig. 12C) largely as in the male, but with En4 in T3 even more obliquely inserted on the tip of En3.

Posterior part of body (Fig. 12D) with CR (“organ fourchu” in Rome and De Deckker 1977) composed of an elongated ramus, ending in bifurcation, with one short, bluntly pointed branch and one longer, hook-like branch; one additional caudal lobe set with pseudochaeta.

#### Measurements.

See Table 2.

**Table 2.** Measurements of Recent and fossil species of *Cytheridella* (from literature) and from specimens of *C.whitmani* used in the present paper for illustration by SEM (all in µm). F = female. M = Male. FOSS = Fossil. Wrozyna et al. (2014) identified two female morphotypes (large F and small F). Victor (1987) reported on two different populations of *C.tepida*, one from northern and one from southern Nigeria, with large size differences between them. “/” = measurements not given in the reference.

Literature Data				
Species	L	H	W	References
*C.ilosvayi* F	960–1140	910	770	Purper 1974
*C.ilosvayi* M	880–950	550	540	Purper 1974
*C.ilosvayi* large F	1110–1140	/	/	Wrozyna et al. 2014
*C.ilosvayi* small F	920–990	/	/	Wrozyna et al. 2014
*C.monodi* F	760	380	480	Klie 1936
*C.monodi* M	650	350	370	Klie 1936
*C.chariessa* F	870	430	590	Rome and De Deckker 1977
*C.chariessa* M	780	390	440	Rome and De Deckker 1977
*C.damasi* F	900	450	630	Klie 1944
*C.damasi* M	700	380	380	Klie 1944
*C.tepida* North Nigeria F	1140–1160	570–590	780–810	Victor 1987
*C.tepida* North Nigeria M	950–1000	540–550	550–570	Victor 1987
*C.tepida* South Nigeria F	1180–1640	590–840	780–820	Victor 1987
*C.tepida* South Nigeria M	1000–1500	540–800	/	Victor 1987
*C.danielopoli* (FOSS)	880	/	/	Danielopol et al. 2023
*C.martingrossi* (FOSS)	1020–1120	/	/	Danielopol et al. 2023
Measurements *C.whitmani* in the present paper				
	L	H	W	
Females				
*C.whitmani*	789		541	INV323006 CpD
*C.whitmani*	768		541	INV323007 CpV
*C.whitmani*	766	453		INV323008 CpRL
*C.whitmani*	768	456		INV3230013 LVi
*C.whitmani*	770	451		INV3230013 RVi
*C.whitmani*	773	449		INV3230014 LVi
*C.whitmani*	775	443		INV3230014 RVi
Males				
*C.whitmani*	683		366	INV323002 CpD
*C.whitmani*	678		349	INV323003 CpD
*C.whitmani*	698	403		INV323004 CpRL
*C.whitmani*	706	425		INV323009 LVi
*C.whitmani*	698	416		INV323009 RVi
*C.whitmani*	689	401		INV323005 LVi
*C.whitmani*	682	390		INV323005 RVi

#### Ecology.

The species is abundant in the permanent lakes on Cape Cod. It occurs on different types of sediments with detritus and was mostly found at ca 0.5–1 m depth.

#### Differential diagnosis.

This species is especially characterised by the shape of the Cp and of the DL and the cop of the Hp, by which it can be distinguished from all living *Cytheridella* species. The selvage is more inwardly displaced than in other species, especially so in the postero-ventral corner of the RV of both genders, which also allows distinction from fossil species. *Cytheridellawhitmani* can be further distinguished from *C.ilosvayi* by the fact that it is significantly smaller (female length approximately 800 µm against 1000 µm or more in *C.ilosvayi*), by the less widely developed posterior brood pouch in the female, and by the fact that the setae on the rimmed pores on the posterior inner flanges (named peripheral marginal infold (pmi) by Danielopol et al. 2023) are simple, whereas these are bi- or multifurcated in *C.ilosvayi*. In addition, the valves and CpRL in males and females of *C.whitmani* have a straight dorsal margin over more than half the length, unlike in *C.ilosvayi* where this margin is curved. The fossil species, *C.martingrossi* Danielopol & Piller, 2023 (in Danielopol et al. 2023), is significantly larger than the new species (female length approximately 1100 µm), and the shape of the CpRL is different in that it is posteriorly upturned. Females of *C.tepida* are between 1100 and 1640 µm long, and as such are the largest species in the genus, much larger than *C.whitmani*. *Cytheridelladamasi* (syn. *C.chariessa*), *C.monodi*, and the fossil *C.danielopoli* Purper, 1979 are of similar sizes as *C.whitmani*, but have different valve shapes. Whereas the dorsal margin in males and females in *C.whitmani* is straight and running parallel with the ventral margin, the dorsal margin in *C.monodi* is curved, while in *C.damasi* it is sloping towards the posterior side. In *C.danielopoli*, the shape of the male valves in inner view resembles that of *C.whitmani*, but the female brood chamber in the latter species is much more developed than in *C.danielopoli* in both lateral and dorsal view (see Purper 1974: pl. 7, figs 23, 24).

## ﻿Discussion

### ﻿Taxonomy of *Cytheridella* species

Swain and Gilby (1964) described *Metacyprisometepensis* from Lake Nicaragua, but in a rather incomplete way. For example, even though they found males, they did not describe the Hp, and what they called the “third leg of male” does not have the typical “cleaning limb” morphology, so is most likely an illustration of the T2 (1964: fig. 3(2)). However, the carapace shape of both males and females is typical of *Cytheridellailosvayi*, as is the length of the female carapace of approximately 1 mm. This brought Martens and Behen (1994) to transfer this species to the genus *Cytheridella* and to subsequently sink *M.ometepensis* into the synonymy of *C.ilosvayi*.

Tressler (1939) described *Onychocytherealosa* Tressler, 1939 on a single male and single female, both retrieved from the stomach of a fish, a specimen of the American shad, *Alosasapidissima* (Wilson, 1811), caught at Welaka (Florida, USA) in the St. Johns River. Four other ostracod specimens were in the same stomach, which Tressler identified as *Cypriaophthalmica* (Jurine, 1820). As the American shad is an amphidromous, migratory species, Tressler assumed that the cytherid specimens had been eaten in a marine environment, while the specimens of *C.ophthalmica* would have been consumed in freshwater. However, both the incomplete drawing of the female Cp and the accurate drawing of the Hp show that the species is identical to *C.ilosvayi*. Therefore, Pinto and Sanguinetti (1962) synonymised *Onychocythere* with *Cytheridella* and subsequently Cohuo et al. (2017) synonymised *C.alosa* with *C.ilosvayi*.

Ferguson (1967) described *Gomphocythereargentinensis* from Argentina, and illustrated the female Cp and the Hp, which were clearly identical to those of *C.ilosvayi*. Karanovic (2009) sank the former species into the synonymy of the latter.

Danielopol et al. (2018) argued that there were few, if any, differences between *C.ilosvayi* and poorly illustrated *C.boldii* Purper, 1974 and suggested that the latter might be a synonym of the former. We here confirm this opinion.

Finally, *Cytheridellaamericana* was described by Furtos (1936) from the cenotes of Yucatan (Mexico) as *Metacyprisamericana* and was transferred to *Cytheridella* by Danielopol (1981, in Colin and Danielopol 1980), based on Furtos (1936: fig. 46) which shows the T3 being transformed into a cleaning limb which is typical of the genus *Cytheridella*. However, the species was described based on a single female and was poorly illustrated, with a single figure of a valve in lateral view and few figures of appendages. We here propose to consider it an “uncertain species” following the procedure described by Müller (1912) and Meisch et al. (2019, 2024). *Cytheridellailosvayi* is thus left as the only living representative of the genus in the Americas. On the other hand, the fossil species *C.danielopoli* (Cenozoic, from the Upper Amazon Basin) and *C.martingrossi* (in Danielopol et al. 2023) (Sucuriju Solimões Formation; late Middle to early Late Miocene; state of Amazônia) show clear morphological differences with *C.ilosvayi*, especially the latter species. Wrozyna et al. (2014) analysed morphological variability of both limbs and valves of a series of males, females, and juveniles of *Cytheridellailosvayi* and concluded (especially based on valve parameters) that there were two morphotypes of females, but a single morphotype in males and in the different juvenile stages. Wrozyna et al. (2014: 1043) wrote: “The presence of two morphologically similar females and only one type of males indicates the coexistence of female morphotypes which may represent either two (cryptic) species or a mixed reproduction population in which parthenogenetic and sexual reproduction coexists.” In later papers, Wrozyna et al. (2016, 2018, 2019) further confirmed the existence of different morphotypes within *Cytheridellailosvayi*. These morphotypes could in time be allocated formal taxonomic status, in some cases even with separate geographical distributions. But none of the formal species synonymised above could be allocated to such morphotypes, as either the type material is non-existent, or damaged (e.g., Furtos 1936; Tressler 1939). We thus face the paradox that morphotypes might need to become taxonomically formalised, but that at the same time potential names from the past must be excluded.

**Figure 13.**
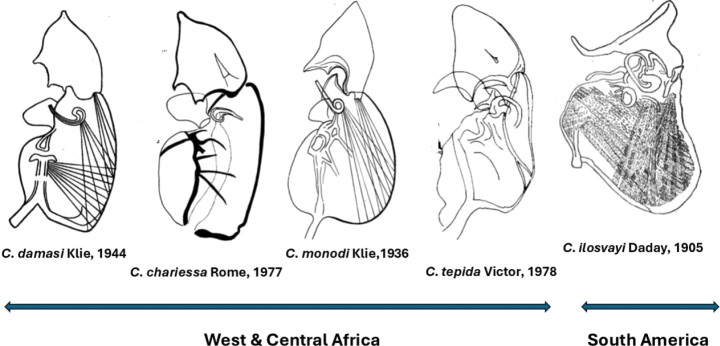
Hemipenes of Recent species of *Cytheridella*. Drawing of *C.ilosvayi* from Purper (1974). All others by original authors. *Cytheridellachariessa* is a synonym of *C.damasi*.

Karanovic (2009) formally synonymised *C.chariessa* (in Rome and De Deckker 1977) with *C.damasi* (in Klie 1944), a synonymy which was already foreshadowed by Victor (1987: 900): “*Cytheridellachariessa* and *C.damasi* are morphologically very similar”. After that, only three African species remained in *Cytheridella*: the said *C.damasi* from Congo, *C.monodi* from Cameroon, and *C.tepida* from Nigeria. These species differ from the South American type species, from *C.whitmani* and from each other mainly by the shape of the DL of the Hp, which is more rectangular, and not as widely triangular as in *C.ilosvayi* (Figs 6D, 13).

### ﻿Comparative morphology

#### ﻿Morphology of the T3

The five extant species (two American and three African) share several synapomorphies, of which the female Cp, with the largely inflated brood pouch, and the modified T3 as cleaning limb in both males and females, are especially notable. Yet, the actual chaetotaxy of the T3 differs between some of these species, and Danielopol et al. (2018) stressed the need to compare the chaetotaxy of this limb in the different (extant) species of *Cytheridella*. In both males and females of the African *C.damasi* and *C.tepida*, T1 and T2 have separate endopodal segments En1–En3, while segment En4 is fused with the end claw h2, but where it remains visible as a swollen base of the claw and the occasional presence of a vestigial setula. In the T3 of these species, segment En4 is also fused with the endclaw h2, but much more visible as a swollen base and it carries a spine-like structure, which we here interpret as being homologous to seta h3. Klie (1936) did not illustrate the thoracic legs of his *Cytheridellamonodi* from Cameroon, but indicated that the three legs are largely similar, with “some exceptions”, from which he cited the presence of an additional hook-like structure at the basis of the end claw in T3. In his key to the genera, he cited for *Cytheridella* “Endklaue mit Sporn” (endclaw with spur – Klie 1936: 307). We can thus assume that the basic chaetotaxy of this leg is similar in all three African species. In *C.ilosvayi* and *C.whitmani*, the situation in both males and females is largely similar to that of the African species, but the fused segment En4 is far less visible at the base of the claw in T1 and T2, while segment En4 of T3 in this species is clearly separated from, and not fused with, the basis of the claw h2 (Purper 1974). Seta h3 is also spine-like, and clearly inserted on the segment En4, which offers support for its homology to seta h3. Based on this small, but significant, morphological difference (the fully separate segment En4 in T3), the South/ Central American species *C.ilosvayi* and *C.whitmani* on the one hand and the three African species on the other, could form two different clusters, with the African one being the more derived one (because of the extra fusion between En4 and claw h2 in T3). This could indicate that the genus, or its ancestral form, already existed before the continental breakup, resulting in South America on the one hand and (west and central) Africa on the other, and that the lineage is thus older than 65 Myr.

#### ﻿Function of the T3

Different morphologies of limbs are nearly always associated with different functions. In most cytheroid ostracod species, the three pairs of thoracic limbs have similar morphologies and are mostly all regarded as walking legs, although in many cases they can also be seen as a means to cling to the (vegetal) surface in habitats with high energy currents. In most Cypridoidea, the three pairs of thoracopods have very different functions: T1 is heavily involved in mating activities in males, T2 is nearly always a walking leg, while in the family Cyprididae, the T3 is modified into a cleaning leg with a pincer-shaped distal part, suitable to clean the natatory setae of the A1 and A2 (Karanovic 2012; Horne et al. 2002).

The morphological differences between T1 and T2 on the one hand and the T3 on the other in species of *Cytheridella* also indicate a different functionality. T3 is more reflexed and, together with the spine-like h3, forms quite a different limb as compared to T1 and T2. The reflexed aspect of the distal part of T3 is more pronounced in the African species, as it starts with the skewed position of segment En2 on En1 and continues with the almost fully reflexed claw h2 with En4 fused to it base. In *C.whitmani*, segments En1 and En2 are in an almost straight (not skewed) position, but En4 and the claws h2 are also fully reflexed (almost 180°). This lead Colin and Danielopol (1980) to interpret the T3 in the species of this genus as a clasping organ, developed to attach the animal to (floating) vegetation, which is very common in Brazilian floodplains, such as Paraná, Pantanal, and Amazon (for example in the genera *Eichhornia*, *Pistia*, *Salvinia* – see Higuti et al. 2007). Here, we interpret the T3 as a functional cleaning limb, where spine h3, the lateral side of En3, and the expanded dorsal tip of En2 form a pincer-like structure, functionally similar, but not homologous, to the pincer-shaped tip of the T3 in most species of Cyprididae.

#### ﻿Caudal ramus

The caudal ramus in cytheroids, unlike in most Cyprididae, is mostly reduced to a relatively simple structure, mostly consisting of some setae. But there are notable exceptions, such as for example in species of the genus *Gomphocythere* Sars, 1924, where the posterior part of the female abdomen comprises two complete caudal rami, each consisting of two setae and three hirsute lobes, while a single furcal organ (FO) is situated dorsally on the abdomen, close to what is assumed to be the caudal seta (CS). In male *Gomphocythere*, the CR consists of one or two simple setae, incorporated in the proximo-ventral part of the Hp (Martens 2003). Regarding the species of *Cytheridella*, the presence/absence and shape of the CR is unclear. Klie (1936) wrote, for both sexes of *C.monodi*, “Eine Furka ist nicht vorhanden” (There is no furca). Klie (1944: fig. 58), on the other hand, illustrated a stout, distally bifurcated rod in the female as “furka” but did not mention it for the male. Rome and De Deckker (1977: pl. 8, fig. q) called this the “organ forchu” in female *C.chariessa*, where it is much smaller than in *C.whitmani* and again, mentioned no CR for the male. For female *C.tepida*, Victor (1987: 898) wrote “Caudal process blunt, devoid of furcal rami”, while he did not mention it for the male, also not in the fairly detailed description of the Hp (p. 900). Purper (1974: pl. 5, figs 8, 9) illustrated a bifurcated rod (which she called caudal ramus) for females *C.ilosvayi*, but did not mention either furca or caudal ramus in the description of the male. In female *C.whitmani* the CR is a single stout rod, distally bifurcate with the larger distal ramus hook-like and pointed; the two distal rods together make for a pincer. In male *C.whitmani*, the CR consists of two stout setae, one of each situated at the base of the Hp, but not fused with it. It is therefore not possible at this stage to interpret the presence/absence and shape of the CR in this genus in a phylogenetic context: were the CR in female *C.monodi* and *C.tepida* missed during the original description or are they really absent in these species? Is *C.whitmani* the only species with CR in the male, or were they missed in all other species of this genus? Re-examination of the type materials of the other species could provide the answers.

#### ﻿Hemipenis

For comparative purposes, the Hp of the other extant species of *Cytheridella* are illustrated in Fig. 13. This figure shows that *C.ilosvayi* from South and Central America has an aberrant DL on the Hp, while the DL of *C.whitmani* is more in line with those of the African species. However, the African species have a well-developed lower ramus, which appears to be absent (*C.whitmani*) or is much smaller (*C.ilosvayi*; see Wrozyna et al. 2018: fig. 4) in the Neotropical species. Fig. 13 also offers support for the synonymy of *C.chariessa* with *C.damasi*, as the Hp of both species is almost identical.

#### ﻿External valve morphology

The external valve ornamentation in both males and females is complex and highly developed. Almost the entire external surface of the valves is covered with pits, mostly organised in circular (anterior and posterior) or random (central parts) patterns. In *C.ilosvayi*, these pits are just shallow and closed indentions. In *C.whitmani*, several of these pits contain what looks like incompletely developed sieve-type pores, although for most of these it is difficult to see as they are cluttered with sticky dirt. A complete sieve-type pore as illustrated by Danielopol et al. (2018) for *C.boldii* was not observed by us in *C.whitmani*, despite the examination of close to 200 SEM images of several male and female carapaces and valves.

The surface of the Cp of the new species carries several rimmed pores, while towards both the anterior and posterior extremities, both setae on conical elevations (so called *Porenwarzen*), as well as long and stiff setae occur. The latter can give the impression that this species is spiny, but these structures are clearly setae and not spines. The term *Porenwarzen* is also used for similar structures in some species of Cyprididae, for example in *Eucyprisvirens* (Jurine, 1820) (see Meisch 2000), but it is uncertain whether these structures are fully homologous in these distantly related ostracod lineages.

#### ﻿Pseudochaeta on valves and upper lip

Both valves in at least *C.ilosvayi* and *C.whitmani* carry internal rows of long and fine setulae on the anterior calcified inner lamella. Danielopol et al. (2023) have called these “cuticular filaments”. These structures do not follow a continuous line, but rather form two different half-rows which meet slightly below the middle. The top half row is situated more distally, the bottom one more proximal to the inner margins. These rows do not seem to be associated with inner lists of vestigial selvages, and their origin (and function) remains unclear. In species of *Herpetocypris* Brady & Norman, 1889 (Cyprididae) one or two ancient inward displacements of selvages have left the anterior calcified lamellae with rows of setae (Gonzalez Mozo et al. 1996), but these are clearly associated with (ancient) marginal selvages, which is not the case here. Daday (1905) figured the upper lip of *C.ilosvayi* with distal filaments, probably pseudochaetae. A similar illustration appears in Rome and De Deckker (1977: pl. 8I) for *C.chariessa*. Also, Colin and Danielopol (1980: fig. 11D) illustrated this type of labrum for *Cytheridella* sp. from Los Palacios, Cuba, which has long and dense pseudochaetae distally. One could wonder if the pseudochaeta on the upper lip and those on the valves are interacting with each other, perhaps during (filter) feeding. This will be described and discussed for several non-marine ostracod species elsewhere.

### ﻿Status of *C.whitmani* in Cape Cod

The African *Cytheridella* species are thus far known from tropical Africa only (Cameroon, Congo, and Niger), while *C.ilosvayi* occurs in the (sub-) tropical regions of South and Central America. However, in Cape Cod, *C.whitmani* survives in a climate with maritime influence, with warm summers and cold winters. Cushman (1907), Sharpe (1910), and especially Furtos (1935) dealt with species collected from localities on Cape Cod, several of which are situated close to Woods Hole, Falmouth, Barnstable, and East Sandwich. Those are the same places from which *C.whitmani* was collected in large numbers during the present survey (in 20 of 24 sampled lakes – see above). However, whereas these older papers together reported 21 species of ostracods (Table 1), none mentioned ostracods that would even remotely resemble a species of *Cytheridella*. It is of course possible that the species was missed during the sampling efforts in the first half of the 20^th^ century on which these papers report. However, as a case in point, one of the localities from which Furtos (1935) described ostracods, is “Marston Mills Pond”. There are three likely candidates from this locality (presently called Mystic Lake, Middle Pond, and Hamblin’s Pond) and *C.whitmani* presently occurs in all three of these lakes (see above). Therefore, *C.whitmani* could be considered an invasive species in the Cape Cod peninsula and arrived there after 1935. This hypothesis can be tested by analysing cores from lakes which now carry the species in abundance. There could be a link between this presumed recent and successful invasion and the fact that winters are becoming less cold in the peninsula (Valiela and Bowen 2003).

## Supplementary Material

XML Treatment for Timiriaseviinae

XML Treatment for Cytheridellini

XML Treatment for Cytheridella

XML Treatment for Cytheridella whitmani

## ﻿Additional information

### Author ORCIDs

Koen Martens https://orcid.org/0000-0001-8680-973X

Nadiny Martins de Almeida https://orcid.org/0000-0002-4380-125X

Michael Shribak https://orcid.org/0000-0002-5849-6294

Janet Higuti https://orcid.org/0000-0002-3721-9562

Isa Schön https://orcid.org/0000-0001-9269-6487

### Data availability

All of the data that support the findings of this study are available in the main text.
